# Climate change, urban health, and the promotion of health equity

**DOI:** 10.1371/journal.pmed.1002621

**Published:** 2018-07-24

**Authors:** Jerald A. Fagliano, Ana V. Diez Roux

**Affiliations:** Dornsife School of Public Health, Drexel University, Philadelphia, Pennsylvania, United States of America

## Abstract

Jerald Fagliano and Ana Diez Roux discuss the challenges of climate change in an urban environment, but also opportunities for healthier lifestyles and green spaces.

A core principle of public health practice is the obligation to empower and protect the most vulnerable populations [1]. This is closely related to the public health goal of eliminating health inequities. The Intergovernmental Panel on Climate Change (IPCC) has pointed out that climate change risks to health will be distributed unevenly, with some population groups being more likely to suffer the adverse consequences than others [2].

Urban populations, and especially socially and economically disadvantaged populations within urban areas, are likely to be especially vulnerable to the adverse effects of climate change [3]. For example, it is well known that people living in urban centers are exposed to higher extreme temperatures because of the heat island effect. Urban populations may have concurrent exposures to air pollutants and social stressors, and those with low income may be more likely to suffer from comorbid conditions that make them more sensitive to heat effects. Low-income populations are also less able to take adaptive actions, for example, because they lack and cannot afford air conditioning to avoid heat exposure [4]. Low-income, urban neighborhoods may have less green space, less energy-efficient housing, and fewer community resources necessary to escape and mitigate the impact of heat. They may also live in areas that are more subject to flooding and have fewer resources to adjust to flooding when it occurs [5]. The growth of urban populations all over the world makes a focus on the impact of climate change on health in cities especially relevant.

Public health practitioners have long acted based on a recognition that the health of the population is inextricably linked to protection of the environment and the long-term sustainability of human interactions with the environment. This interdependence of the natural and built environments is a foundational belief in public health [1]. These connections are manifested most recently in the emerging planetary health movement. Integral to planetary health is the notion that interventions intended to protect the health of ecosystems will often result in positive impacts on population health, which may be viewed as “co-benefits” [6].

The recognition of health co-benefits is critical to public health efforts to prevent health impacts of climate change and is fundamental to the public health goal of eliminating health inequities. Health co-benefits of policies to mitigate climate changes may be realized in the short term, while the environmental benefits may be apparent only after many years or decades [7]. There are many ways in which environmental interventions intended to prevent or mitigate climate change may have important health co-benefits for urban populations.

Shifts away from fossil fuel combustion toward renewable and noncarbon sources to power the transportation and electricity-generation systems, together with improvements in efficiency and other types of energy conservation, will reduce emissions of carbon dioxide and slow the rise in temperatures, as intended [2,8,9,10,11]. This shift will also result in a reduction in emissions of other air pollutants from these energy sources, positively affecting air quality and yielding a substantial health co-benefit [11,12,13], and much of the benefit will likely accrue to urban populations, where air pollution may be worse and large numbers of people live and work [3].

Investments in urban designs that encourage active modes of travel will not only reduce carbon dioxide emissions and improve air quality but could also result in benefits to cardiovascular health from increased physical activity and reduced obesity [9,11,14,15]. An American Heart Association statement notes that policies to promote active transport increase overall physical activity through the life span [16]. Safe, active transport design that separates cars from bicyclists and walkers could have the added health co-benefit of reducing the frequency and severity of injuries. These co-benefits could be most impactful among urban, low-income populations, who are more likely to have lower physical activity and a higher prevalence of obesity, diabetes, and cardiovascular disease [3].

Considering their total energy use, buildings account for 32% of global greenhouse gas emissions [17]. Climate mitigation investments made in neighborhoods to promote conservation and improve energy efficiency of housing, through improved insulation and air-tightness of buildings, will not only reduce energy consumption and carbon dioxide emissions but can also serve as adaptive actions by reducing vulnerability to both cold and hot extremes in temperature [9,18].

Greening of urban areas could reduce the urban heat island effect, contribute to energy conservation by reducing the need for energy for cooling, and potentially improve local air quality. Co-benefits from neighborhood greening could include health benefits from increased nature contact: enhanced well-being; reduced stress, aggression, depression, and anxiety; and increased physical activity from recreational opportunities in green spaces in urban environments [11,19]. In many urban areas, opportunities for the health benefits of greening are larger in low-income communities [20].

Although discussions of co-benefits are often framed around health co-benefits of actions to mitigate or adapt to climate change, it is useful to recognize that some public health actions that have long been promoted in urban areas may be seen as having co-benefits in the climate arena. For example, more widespread adoption of dietary changes promoted to improve cardiovascular health, such as lower consumption of meats (particularly beef), would substantially reduce greenhouse gas emissions because production systems that support diets high in meat generate much more emissions than those that support vegetarian or high fish diets [11]. Likewise, campaigns whose purpose is to promote health through increased physical activity such as biking and walking will have important environmental co-benefits through reductions in automobile-related pollutant emissions. These health policies thus have the potential for doubly benefiting the most disadvantaged, through direct health impacts and through their secondary impacts via environmental change and prevention of the most adverse effects of climate change.

The public health impacts of climate change may be the largest global health challenge in the current century. Climate change is occurring in the context of rapid urbanization. Urban areas present many challenges in terms of addressing the health consequences of climate change, but they also provide many opportunities. Urban policies that promote dense, walkable, and transit-friendly communities have multiple health and environmental co-benefits [21]. Strategies to reduce the consumption of animal products and processed foods in urban settings, through taxation, for example, will also have both types of co-benefits. These co-benefits are likely to be magnified for disadvantaged urban populations, given their greater exposure to environmental hazards and their adverse health effects.

Health, the environment, and equity are closely entwined. This close interrelation is especially critical in growing urban areas. Designing, managing, and governing urban areas so that they are simultaneously healthier, environmentally sustainable, and more equitable is a critical need and also an opportunity for the future of population health and our planet.
